# Permeability and Selectivity of PPO/Graphene Composites as Mixed Matrix Membranes for CO_2_ Capture and Gas Separation

**DOI:** 10.3390/polym10020129

**Published:** 2018-01-29

**Authors:** Riccardo Rea, Simone Ligi, Meganne Christian, Vittorio Morandi, Marco Giacinti Baschetti, Maria Grazia De Angelis

**Affiliations:** 1 Dipartimento di Ingegneria Civile, Chimica, Ambientale e dei Materiali (DICAM), Università di Bologna, Via Terracini 28, 40131 Bologna, Italy; riccardo.rea3@unibo.it (R.R.); marco.giacinti@unibo.it (M.G.B.); 2 Graphene XT s.r.l., 40131 Bologna, Italy; simone.ligi@graphene-xt.com; 3 CNR-IMM Section of Bologna, via Gobetti, 101-40129 Bologna, Italy; christian@bo.imm.cnr.it (M.C.); morandi@bo.imm.cnr.it (V.M.)

**Keywords:** graphene, membranes, gas separation, CO_2_ capture, permeability, selectivity, PPO

## Abstract

We fabricated novel composite (mixed matrix) membranes based on a permeable glassy polymer, Poly(2,6-dimethyl-1,4-phenylene oxide) (PPO), and variable loadings of few-layer graphene, to test their potential in gas separation and CO_2_ capture applications. The permeability, selectivity and diffusivity of different gases as a function of graphene loading, from 0.3 to 15 wt %, was measured at 35 and 65 °C. Samples with small loadings of graphene show a higher permeability and He/CO_2_ selectivity than pure PPO, due to a favorable effect of the nanofillers on the polymer morphology. Higher amounts of graphene lower the permeability of the polymer, due to the prevailing effect of increased tortuosity of the gas molecules in the membrane. Graphene also allows dramatically reducing the increase of permeability with temperature, acting as a “stabilizer” for the polymer matrix. Such effect reduces the temperature-induced loss of size-selectivity for He/N_2_ and CO_2_/N_2_, and enhances the temperature-induced increase of selectivity for He/CO_2_. The study confirms that, as observed in the case of other graphene-based mixed matrix glassy membranes, the optimal concentration of graphene in the polymer is below 1 wt %. Below such threshold, the morphology of the nanoscopic filler added in solution affects positively the glassy chains packing, enhancing permeability and selectivity, and improving the selectivity of the membrane at increasing temperatures. These results suggest that small additions of graphene to polymers can enhance their permselectivity and stabilize their properties.

## 1. Introduction

The removal of CO_2_ from gaseous streams produced in energy production and energy-intensive industrial processes is one of the most straightforward ways to reduce the global warming effect due to atmospheric CO_2_ increase [1,2].

Membrane separations are considered suitable, and environmentally friendly, technologies to capture CO_2_ both in post combustion (removal of CO_2_ from N_2_) and pre combustion (removal of CO_2_ from H_2_) [3,4,5,6]. Mixtures of H_2_ and CO_2_ are generated in the production of hydrogen from steam reforming or biomass gasification, and their purification is necessary not only for environmental aims, i.e., for the reduction of the CO_2_ emitted in the atmosphere, but also for technological purposes, i.e., the purification of H_2_ for use as a fuel or chemical. Most of those processes are carried out at high temperature, and a high-temperature purification process would allow exploiting the thermal level of the gas stream [4].

In membrane processes, the main way to optimize the separation is to use highly permeable and highly selective membrane materials. However, as pointed out in the literature, it is often not possible to simultaneously increase the permeability and the selectivity, which are governed by a tradeoff mechanism [7].

Poly(2,6-dimethyl-1,4-phenylene oxide) (PPO) is a glassy polymeric material suitable for membrane-based gas separation, due to a high permeability and moderate selectivity [8]. In particular, PPO has a H_2_-selective behavior, when exposed to mixtures of H_2_ and CO_2_, i.e., it exhibits a size-sieving ability, because hydrogen has a smaller kinetic diameter than CO_2_ (0.29 vs. 0.33 nm) [9]. The reported ideal H_2_/CO_2_ selectivity value for PPO is 1.5 at room temperature, while an economically feasible membrane separation process would require higher values [4]. However, this material is stable up to 200 °C and H_2_/CO_2_ membrane separation performances are known to be significantly increased by increasing temperature [4]. Thus, PPO results to be an interesting candidate for the high temperature separation of this mixture.

A further strategy to enhance the selective behavior of the polymer can be based on the addition of nanosized fillers to the polymer matrix: in particular, nanofillers can modify the chain packing of the polymer, increasing its selectivity. It was reported by many authors that separation performance of polymeric materials can be improved by the addition of nanosized particles of different shapes, such as nanospheres [10,11,12,13,14,15,16] and nanotubes [17,18,19]. Nanometric fillers tend to adjust the chain packing of glassy polymers in a way that affects positively the permselectivity, without creating non selective voids, but rather creating additional selective free volume [20].

Graphene is a nanosized material that has been just recently applied to the field of gas separation membranes, as initially it was mostly considered for improving the barrier effect [21,22,23,24,25,26,27,28]. Indeed, it was noticed that, while a defect-free graphenic layer is virtually impermeable to all molecules, some production techniques, such as chemical vapor deposition (CVD), may introduce a microporosity. Moreover, when the graphene is applied in subsequent layers, permeable channels caused by imperfect adhesion may form. Graphene oxide (GO), on the other hand, naturally contains defects induced by the oxidation process, and is endowed with an intrinsic gas permeability and selectivity. Several studies report interesting results of the application of GO in gas separation [29,30,31].

Some other works evaluated the combination of graphene, obtained by direct exfoliation of graphite in polymeric solution, to a polymer of intrinsic microporosity, PIM-1. The studies indicate that the optimal concentration of graphene to maximize the CO_2_ permeability of PIM-1 is 0.1 wt %, and molecular simulations indicate that the presence of graphene nanolayers modifies the polymer distribution [32,33,34].

The addition of 1 wt % of monolayers of GO slightly enhances the gas permeability of poly(trimethyl silyl propyne) (PTMSP), and the selectivity for the couple CO_2_/He and CH_4_/He [35]. The addition of a few layer graphene in the same amount to PTMSP lowers slightly the gas permeability, with factors that increase with decreasing molecule size, and enhances the ideal selectivity for the couples CO_2_/He and CH_4_/N_2_, CH_4_/He. The addition of this filler mainly lowers the CO_2_ diffusivity, leaving the gas solubility unaltered, while the solubility and diffusivity are both enhanced by addition of GO (lateral dimension 2.0 m, thickness 1.1 nm). Multiple layer graphene (lateral dimension 0.2 m, thickness 2–20 nm) lowers the permeability of PTMSP to a significant extent (up to 30%), with a weight fraction of just 1 wt % [35].

Both GO and few-layer graphene allow to impair the aging process of PTMSP, as tracked with He, N_2_, CH_4_ and CO_2_ permeability. Graphene platelets, due to their high aspect ratio, act as physical barriers to the rearrangement of polymer chains, and the diffusion of free volume domains, which cause the ageing. Such explanation is supported by the fact that ageing is mostly reduced by the fillers that have the higher aspect ratio [35].

In view of the previous findings, in this work, we studied whether the separation performance of PPO with respect to some gas mixtures, can be improved by addition of graphene nanoplatelets. Furthermore, in the aim of assessing the effect of temperature on permselectivity, we tested the membranes performance to temperatures as high as 65 °C.

## 2. Experimental

### 2.1. Preparation of Membranes

The solid polymer PPO was purchased by Sigma Aldrich, and chloroform (purity >99.5% Sigma Aldrich, St. Louis, MO, USA) was used as solvent. It is an amorphous material with a rather high *T*_g_ (213 °C), that makes it suitable for use in high temperature separations. The melting point is 268 °C and the density at 25 °C is 1.06 g/cm^3^. The permeability to hydrogen is rather high, and ranges between 87 and 112 Barrer at 25 °C [36,37]; such property is due to a high fractional free volume of the polymer, which is around 18% [8]. In this work, helium is used instead of H_2_ for safety reasons: literature results indicate that the permeability of He is moderately lower than that of H_2_ in this polymer, its permeability ranging between 56 and 75 at 25 °C [8,38]. Therefore, the use of He instead of H_2_ leads to conservative estimates for the H_2_ permeability and H_2_/CO_2_ selectivity.

Two commercial grades of graphene in powder, produced by Graphene XT, were used: Graphene XT6, with a lateral dimension of 5 m and a thickness between 6.0 and 8.0 nm, and Graphene XT7, with a nominal lateral size of 20 m and a thickness of 2 nm.

The procedure to prepare membranes was optimized considering also a previous work [35].

**Figure 1:**
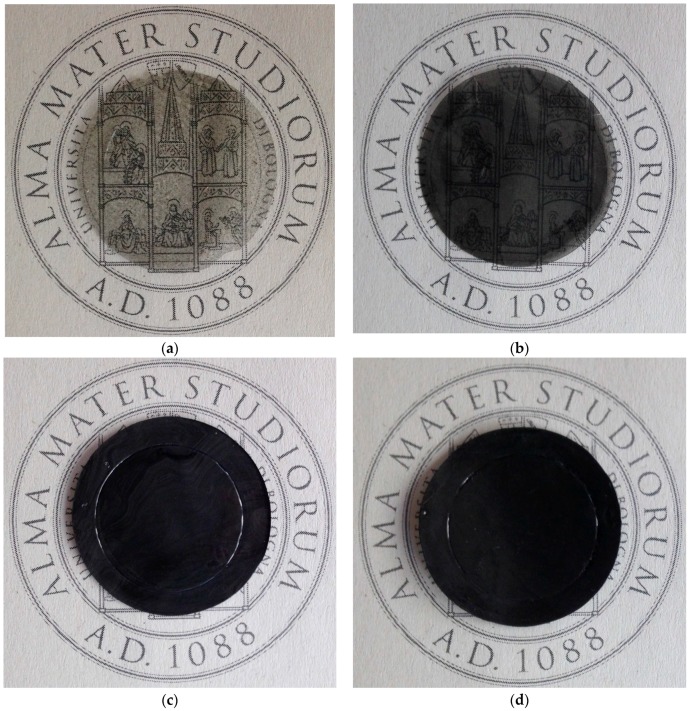
Pictures of the various membranes fabricated: (**a**) PPO + 0.3 wt % of Graphene XT7; (**b**) PPO + 1 wt % of Graphene XT6; (**c**) PPO + 5 wt % of Graphene XT6; and (**d**) PPO + 15 wt % of Graphene XT6.

The polymer was dissolved in chloroform and then graphene powder was added. The resulting suspension was sonicated for at least 15 min, and stirred for one day. In the case of Graphene XT7, the sonication time was increased to 60 min to improve the disaggregation of nanoplatelets, as this materials has a higher aspect ratio than the other filler used. Once the dispersion was complete, the solution was poured on a Petri dish and placed in a clean hood, where the temperature was kept at 50 °C, to ensure fast evaporation of the solvent. The solid film was then removed from the Petri dish, and treated in an oven under vacuum for 1 day at 200 °C. Such treatment not only removes traces of solvent, but also stabilizes the properties of the glassy PPO, with respect to ageing, for a sufficiently long time. It must be noticed, however, that PPO membranes treated at such temperatures show a somewhat lower permeability than untreated ones. Table 1 reports the list of materials produced, the procedure adopted, together with their properties. The photos of the various composite membranes produced are reported in Figure 1: one can notice the increasing darkness of the films with increasing amount of graphene loaded. It must be noticed that a film of pure PPO is completely transparent.

The gases used were N_2_ (SIAD, Ozzano Emilia, Italy, purity of 99.999%), CO_2_ (SIAD, purity of 99.998%) and He (SIAD, purity of 99.9999%).

### 2.2. Permeability Tests

The permeability was measured with a variable pressure apparatus described in previous works [38], by applying an upstream pressure of about 1.4 bar and vacuum on the downstream side. The pure gas permeability at steady state can be calculated from Equation (1), in which ${\frac{dp_{i}}{dt}|}_{s.s.}$ is the slope of pressure versus time curve at steady state, *V_d_* is the calibrated downstream volume, *R* is the universal gas constant, *T* is the system temperature, *A* is the membrane area, *l* is the thickness of the sample and $\left(p_{i}^{up}-p_{i}^{down}\right)$ is the gas pressure difference across the membrane film.

(1) $$P_{i}={\frac{dp_{i}}{dt}|}_{s.s.}\frac{V_{d}}{RTA}\frac{l}{\left(p_{i}^{up}-p_{i}^{down}\right)}$$

The apparatus is placed in a thermostatic chamber which can reach temperatures in the order of 80 °C. Tests were conducted at 35 °C; and for most samples we also carried out tests at 65 °C to investigate temperature effect on permeability of the different gases investigated, i.e., He, N_2_ and CO_2_.

The permeability value is also affected by the uncertainty on the membrane thickness, which depends on the sample considered. The error on ideal selectivity of a single sample however is negligible, because such value is unaffected by downstream volume, permeation area and membrane thickness values as it is clear from Equation (1).

Ideal selectivity for the different gas couple of interest is calculated using Equation (2), which is valid in the case of negligible downstream pressure, as in the tests considered here:

(2) $$\alpha_{ij}=\frac{P_{i}}{P_{j}}$$

where the selectivity is defined as $\alpha_{ij}\equiv \frac{y_{i}^{downstream}/y_{i}^{upstream}}{y_{j}^{downstream}/y_{j}^{upstream}}$. In general, real selectivity can differ from the ideal selectivity, which is estimated using the permeability values performed on the pure gases, but the latter still represents a good indication of the material performance with respect to other membranes as most of the data reported in literature refer to this kind of selectivity [7].

In many cases, we were able also to determine the characteristic time of permeation, i.e., the time required to reach a stable flux across the membrane, the so-called time-lag, *t*_L_, that is related to the gas diffusivity in the membrane by the following relation [39]:

(3) $$D=\frac{1}{6}\frac{l^{2}}{t_{L}}$$

The time lag is actually estimated as the intercept, on the time axis, of the asymptotic straight line representing the downstream gas pressure versus time. It can also be noticed that, due to the phase equilibrium between the gas and the polymer phase, according to which the gas is absorbed onto the polymer surface proportionally to its solubility coefficient *S_i_*, a straight forward relation holds true between the gas permeability, and its diffusivity and solubility coefficient in the polymer:

(4) $$P_{i}=D_{i}S_{i}$$

According to which, the selectivity can be decomposed into a diffusivity-based, and a solubility-based contribution:

(5) $$\alpha_{ij}=\left(\frac{D_{i}}{D_{j}}\right)\left(\frac{S_{i}}{S_{j}}\right)$$

Usually, for a same gas couple, the two contributions are, respectively, lower than unity and higher than unity. In general, the diffusivity-selectivity prevails, so that smaller gases are generally more permeable than larger ones in most polymeric membranes. In some cases, the solubility prevails, as happens for instance when CO_2_ is concerned. For this reason, not all polymeric membranes have a H_2_/CO_2_ selectivity higher than 1, but some of them, due to a very high CO_2_ solubility, exhibit a CO_2_-selective behavior [40].

The temperature dependence of permeability, diffusivity and solubility is governed by Arrhenius-like laws, as follows:

(6) $$P_{i}=P_{i}\left(T_{0}\right)\exp\left(-\frac{E_{P,i}}{RT}\right)$$

(7) $$D_{i}=D_{i}\left(T_{0}\right)\exp\left(-\frac{E_{D,i}}{RT}\right)$$

(8) $$S_{i}\left(T\right)=S_{i}\left(T_{0}\right)\exp\left(-\frac{\Delta H_{S,i}}{RT}\right)$$

where *E_P_* and *E_D_* are the permeation and diffusion activation energies, respectively, which are usually positive for gases in glassy polymers, and Δ*H_S_* is the sorption enthalpy, which is usually negative. Accordingly, the permeability and diffusivity increase with temperature, while the solubility decreases with it.

Due to Equations (4)–(8), the permeation activation energy has a diffusivity and solubility contribution as follows:

(9) $$E_{P,i}=E_{D,i}+\Delta H_{S,i}$$

For the selectivity dependence on temperature is concerned, one can combine Equations (2) and (6) to obtain:

(10) $$\alpha_{i,j}\left(T\right)=\alpha_{i,j}\left(T_{0}\right)\exp\left(-\frac{E_{P,i}-E_{P,j}}{RT}\right)$$

which indicates that the selectivity increases with temperature if the more permeable gas has a higher activation energy than the less permeable one, while it decreases with temperature if the opposite is true. In general, the second situation is more frequent, as less permeable gases have higher activation energies. The couples H_2_/CO_2_ and He/CO_2_ in PPO form an exception, because H_2_ and He are more permeable at room temperature than CO_2_, but also have a higher activation energy of permeation. The activation energy of CO_2_ is low, due to a high contribution of sorption, an exothermic process, on the permeation. Therefore, for H_2_-selective membranes, as the ones of the present paper, increasing the temperature enhances the H_2_ permeability and H_2_/CO_2_ selectivity.

## 3. Results and Discussion

### 3.1. SEM Analysis

**Figure 2:**
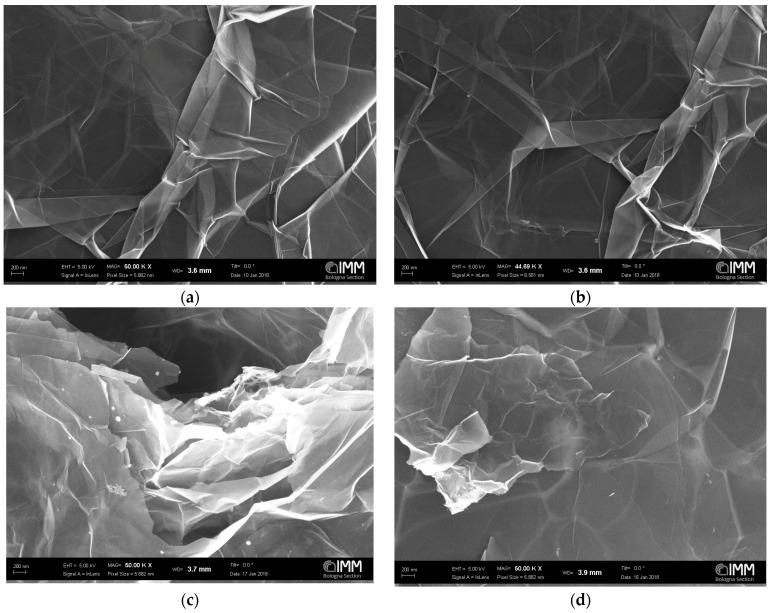
SEM images of: dry Graphene XT7 powder (**a**,**b**); and of the Graphene XT7 dispersed in water: before sonication (**c**); and after a 15 h sonication (**d**).

First, neat graphene in powder was characterized. The Graphene XT7 was chosen for this analysis and two images are reported in Figure 2a,b that show some wrinkles. The effect of dispersing the platelets in water is that of swelling and opening the structure, as shown in Figure 2c,d. Then, the effect of sonication on the sample morphology was analyzed. Pictures of the sample dispersed in water and sonicated for only 10 min exhibit lateral sizes, from the picture analyzed and reported in Figure S1 in the Supplementary Materials, larger than what is declared by the supplier, between 26 and 63 micrometers (based on seven platelets). In Figure S2 in the Supplementary Materials, we report similar pictures taken on a sample of Graphene XT7 (in water suspension) sonicated for 15 h. As can be seen, the platelets analyzed have a lateral size ranging between 34 and 43 micrometers, also based on seven platelets. Therefore, it seems that the sonication produces just a slight decrease of the average lateral size after 15 h, but the size distribution of the platelets becomes narrower.

**Figure 3:**
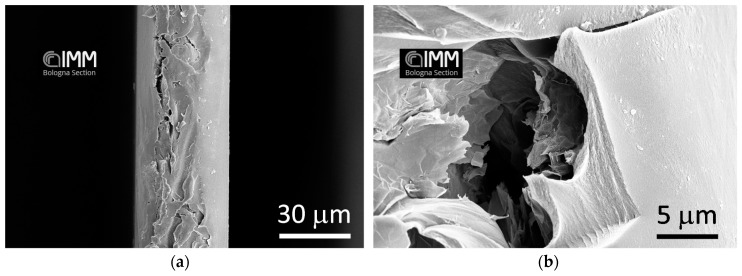

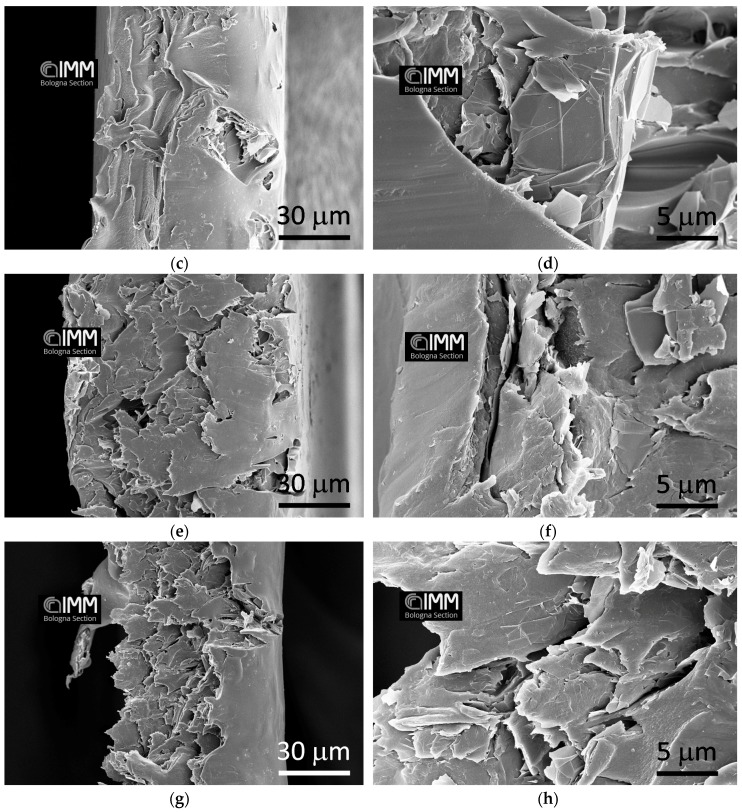
SEM images of membranes of: (**a**,**b**) PPO/0.3 wt % of Graphene XT 7; (**c**,**d**) PPO/1 wt % of Graphene XT 6; (**e**,**f**) PPO/5 wt % of Graphene XT 6; and (**g**,**h**) PPO/15 wt % of Graphene XT 6.

**Figure 4:**
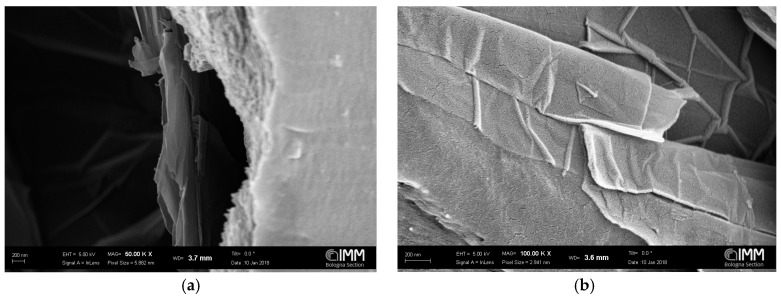

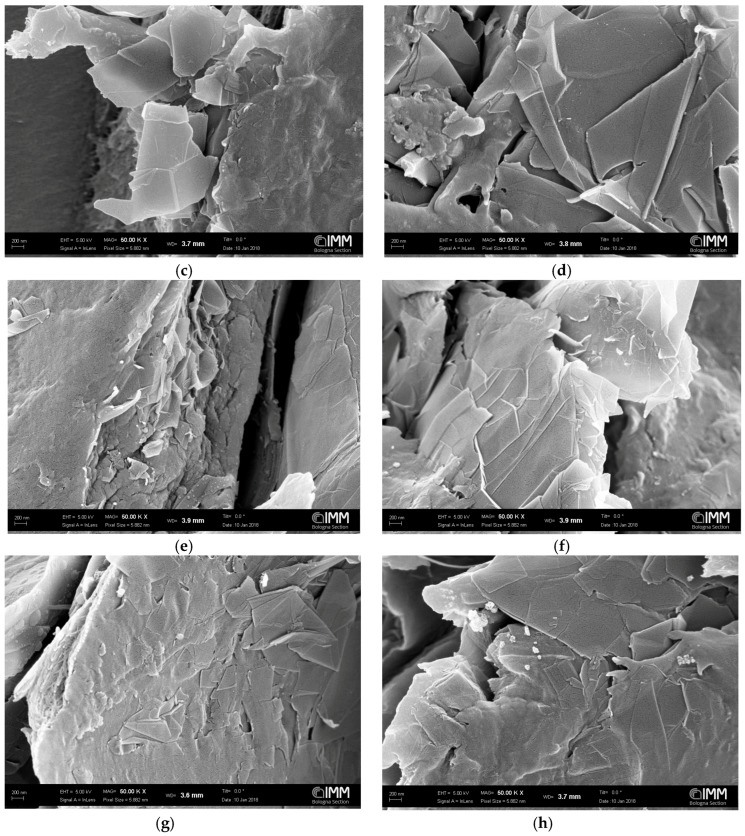
SEM images of membranes of: (**a**,**b**) PPO/0.3 wt % of Graphene XT 7; (**c**,**d**) PPO/1 wt % of Graphene XT 6; (**e**,**f**) PPO/5 wt % of Graphene XT 6; and (**g**,**h**) PPO/15 wt % of Graphene XT 6.

Pictures of the cross section of PPO + graphene membranes are reported in Figure 3. It is apparent that, by increasing the concentration of graphene, the cross section becomes filled with graphenic domains. Those structures are aligned perpendicular to the membrane cross section, thereby increasing the tortuosity of diffusing molecules. The images also show that some voids form at the interface of PPO and graphene. Images with higher magnification were also taken (Figure 4), and show that the surface of graphene platelets remains similar to the one observed in the neat powder (Figure 2).

### 3.2. Permeability

**Figure 5:**
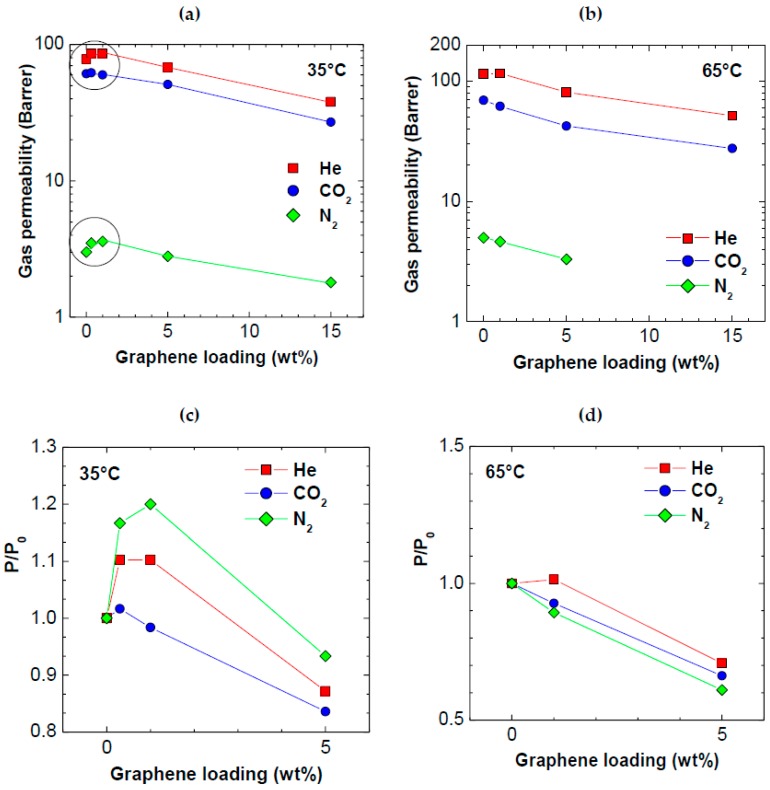
Gas permeability at: (**a**) 35 °C; and (**b**) at 65 °C, as a function of graphene loading in PPO. Variation of PPO permeability after addition of graphene vs. graphene loading at: (**c**) 35 °C; and (**d**) 65 °C.

In Table 2 and Figure 5, we report the permeability values measured at 35 and 65 °C in membranes with increasing loadings of graphene.

It can be seen that, while the addition of small amounts (below 1 wt %) of both types of graphene enhance the He permeability, by about 10%, increasing the filler loading to higher amounts (5 and 15 wt %) reduces the permeability, for all gases considered. This is because the wide graphene platelets, in this range of concentration, significantly increase the tortuosity of gases diffusive path in the membrane. The permeability of CO_2_ seems unaffected by the presence of graphene up to a loading of 1 wt %. The permeability of N_2_, on the other hand, follows a same qualitative behavior as that of helium, increasing by about 20% for graphene loadings lower than 1 wt %, and decreasing for higher values. The permeability enhancement observed could be due to various factors, such as the formation of additional free volume at the interphase between polymer and filler, and the permeation of the gas through graphene layers.

At 65 °C, no increase of permeability could be detected after addition of graphene: we believe that, at this temperature, the transition between the regime where the free volume increase prevails and the one in which the tortuosity increase dominates occurs at very small graphene loadings, or the first regime does not occur at all. At higher temperatures, indeed, the mobility of both diffusing gases and polymeric chains is higher, and the diffusion and permeation process relies less on the presence of intrinsic free volume in the solid matrix.

### 3.3. Selectivity

**Figure 6:**
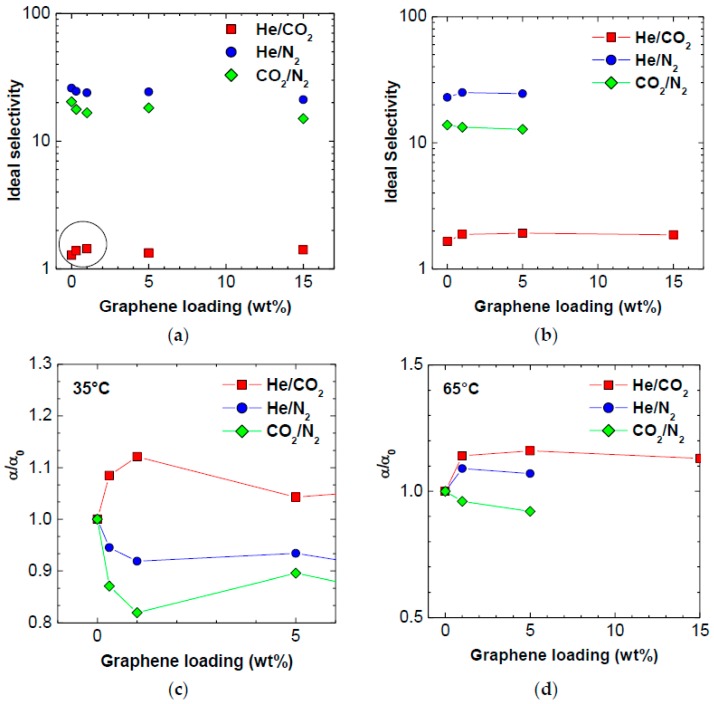
Gas ideal selectivity at: (**a**) 35 °C; and (**b**) at 65 °C, as a function of graphene loading in PPO. Variation of PPO selectivity after addition of graphene vs. graphene loading at (**c**) 35 °C and (**d**) 65 °C.

The ideal selectivity values calculated with Equation (2) at 35 °C are reported in Table 3 and Figure 6. Three types of separations are considered: He/CO_2_, He/N_2_ and CO_2_/N_2_. The first separation (He vs. CO_2_) is favored by high temperatures, i.e., both permeability and selectivity increase with increasing temperature, due to higher activation energy of permeation for He than for CO_2_. The other separations considered, namely He/N_2_ and CO_2_/N_2_ are, in terms of selectivity, not favored by temperature, as the selectivity of polymers for such couples decreases with temperature, due to the unfavorable difference between the permeation activation energies of the two gases (see Equation (10)).

The addition of graphene in all proportions produces an increase of He/CO_2_ selectivity of the polymer at 35 °C, of about 10%. For the He/N_2_ separation, the selectivity values decrease monotonically with increasing graphene content at 35 °C, going from 26.0 to 21.1 at the largest loading. For the CO_2_/N_2_ mixture, the selectivity also decreases monotonically with increasing graphene loading, from 20.3 to 15.0. Such trends are due to the gas-dependent variations of permeability induced by addition of graphene. The gas that experiences the highest increase of permeability after addition of small loadings of graphene is the largest one, namely nitrogen. For samples containing larger amounts of graphene, the smallest reduction of permeability recorded is again that of nitrogen. Thus, both He/N_2_ and CO_2_/N_2_ selectivity decrease. Such trend is attributable mostly to the kinetic term of permeability, the diffusivity, which is strongly affected by molecule size, as will be seen in the following. When the He/CO_2_ selectivity is considered, on the other hand, the size of the molecule plays a less important role, as CO_2_ permeability is also strongly affected by solubility, which depends on the molecule condensability rather than on its size. Therefore, for this couple of gases, the size-sieving capacity of the membrane slightly increases with the addition of graphene, rather than decreasing. When the temperature is increased to 65 °C, the situation slightly changes, in particular for the case of the He/N_2_ mixture, for which graphene addition positively affects the selectivity. The incorporation of filler is also beneficial for the He/CO_2_ separation, similar to the lower temperature case. The CO_2_/N_2_ selectivity, on the other hand, decreases with graphene addition also at 65 °C. The different trends of selectivity at different temperatures are due to the effect that graphene has on the thermal dependence of the gas permeability in the polymer, which we will discuss below.

### 3.4. Diffusivity

**Figure 7:**
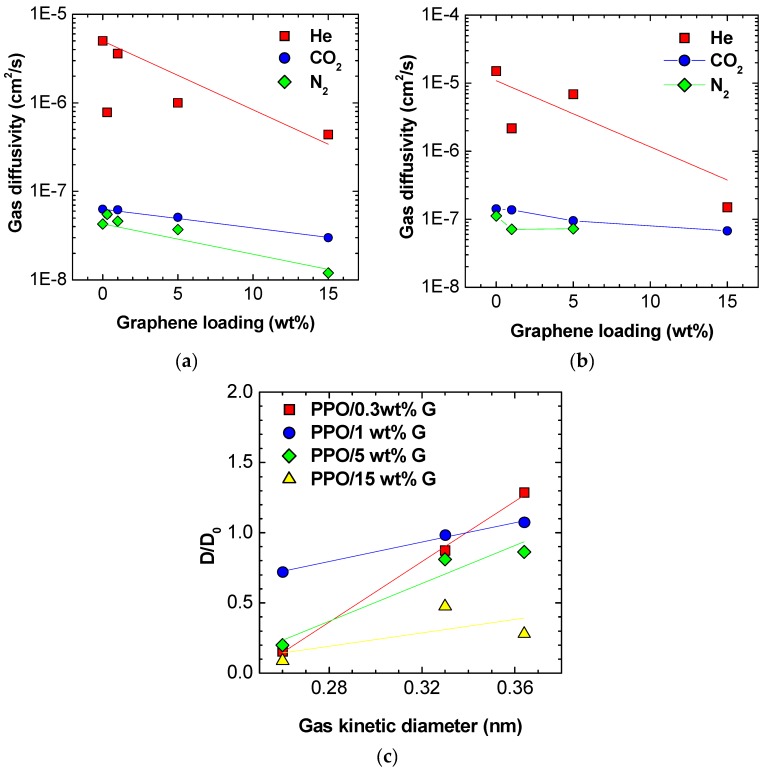
Diffusivity of the various gases in the PPO composite membranes at various values of graphene loadings at: (**a**) 35 °C; and (**b**) 65 °C; (**c**) Gas-dependent effect of graphene on membrane diffusivity at 35 °C: the chart shows the relative increase of membrane gas diffusivity after graphene addition versus the size of the gas molecule for various loadings of graphene.

The diffusivity is a kinetic quantity that contributes to the permeability, according to Equation (4). In particular, diffusivity is strongly affected by variations of the polymer free volume, and by the chain packing of the membrane. Therefore, it is expected to be the quantity that is most strongly affected by the introduction of filler with a peculiar morphology, such as the high aspect ratio graphene platelets with one nanometric dimension. Furthermore, in size-selective polymers such as the one that we are considering, diffusivity dictates the selective behavior, rather than solubility, as happens for instance in the case of higher free volume glassy polymers such as PTMSP and PIM-1. In Table 4, we report the variations observed for the diffusion coefficients as a function of graphene loading. It can be seen that, when high filler loadings are considered, a marked decrease of diffusivity is measured, which is because the graphenic nanoplatelets act as physical barriers and increase tortuosity. At lower loadings, the behavior shows a large scattering. The diffusivity data obtained at 35 and 65 °C are also reported in Figure 7a,b.

It is often seen, in mixed matrix membranes comprising nanometric fillers, that the variations of permeability induced by filler are gas size-dependent [15,16]. Usually, porous fillers with a size selective ability increase more effectively the permeability towards smaller gases, while the opposite can be observed in the case of nanoscopic impermeable fillers such as fumed silica [15,16]. In our previous work, we have found that the addition of graphene reduces more strongly the permeation of small gases, such as Helium, and to a lower extent the permeation of larger molecules, such as Nitrogen [35]. In this work, we observe a similar trend: we reported diffusivity variations after graphene addition versus the kinetic diameter of penetrating molecule in Figure 7c. We see values of *D*/*D*_0_ close to 1 for the larger molecule, namely N_2_, and much smaller than unity for the smallest molecule, Helium.

**Figure 8:**
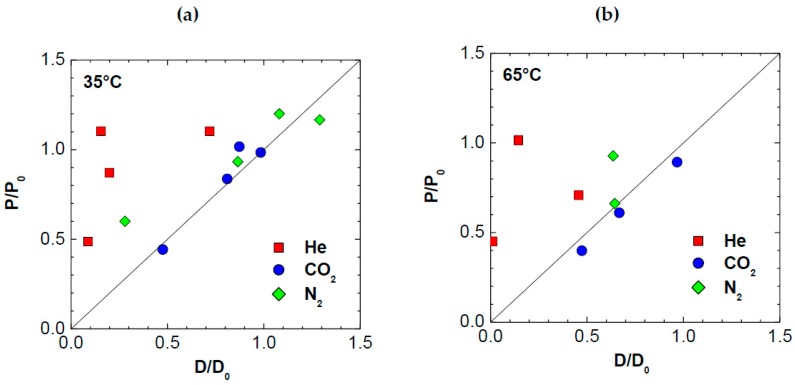
Parity plot showing the correlation between the variation of diffusivity induced by addition of graphene and the corresponding variation of permeability at: (**a**) 35 °C; and (**b**) 65 °C.

To effectively correlate the observed variations in permeability to variations of diffusivity, we plotted a parity curve, reporting the observed variations in permeability after addition of graphene, *P*/*P*_0_, to the ones observed for diffusivity, *D*/*D*_0_, in Figure 8. It can be seen that, while for CO_2_ and, less markedly, for N_2_, the permeability trend is strongly associated to the diffusivity one, confirming that diffusion plays a strong role in such membranes, for He, the behavior is different. For this gas, the permeability of composite membranes is reduced less than the diffusivity by addition of graphene. Although the error associated to the evaluation of the diffusivity from the time lag measurement is larger in the case of Helium, which can cause some significant random scattering, such trend is systematic, i.e., all the data fall above the bisector. If the deviations were due merely to scattering associated to experimental error, they should have lied both above and below the bisector. According to the solution–diffusion model expressed by Equation (4), one should have:

(11) $$\frac{P}{P_{0}}=\frac{D}{D_{0}}\frac{S}{S_{0}}$$

so that, if *P*/*P*_0_ = *D*/*D*_0_, as it approximately happens in the case of N_2_ and CO_2_, one concludes that *S*/*S*_0_ = 1, i.e., that the solubility of such gases is not affected by the presence of graphene. On the other hand, the same equation applied to the case of helium indicates that *S*/*S*_0_ > 1, i.e., that graphene enhances the solubility of helium in the membrane. Although the solubility of helium in solids is generally small in absolute value, we are considering relative variations here, which can be significant. Furthermore, the helium molecule, due to its small size, could be adsorbed onto the graphene layers interface but also between the layers of graphene platelets. This solubility behavior counterbalances the strong reduction of diffusivity observed in the case of helium.

### 3.5. Analysis of Temperature Effect

It is very interesting to analyze the effect of temperature on those composite membranes. Normally, the transport properties of polymers, especially the permeability, are strongly dependent on temperature, and, to our knowledge, this is the first analysis of the temperature effect on transport properties of polymer/graphene composites for separation.

**Figure 9:**
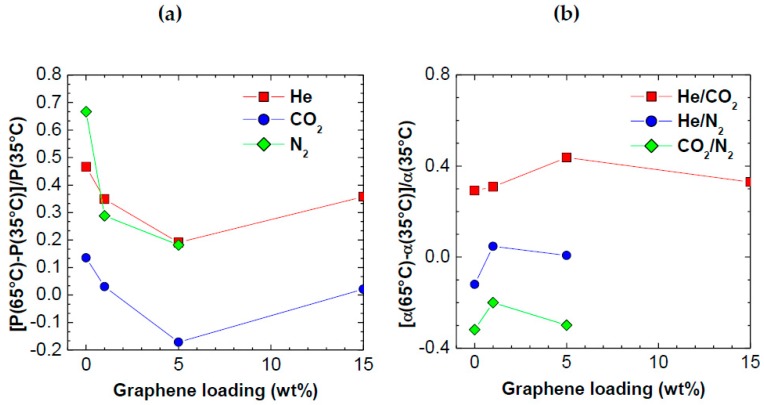
Relative variation of: (**a**) permeability; and (**b**) selectivity with temperature versus graphene loading of different PPO composite membranes.

Figure 9a reports the temperature effect on permeability, as a function of graphene loading, and explains the observed trend of selectivity with temperature. It is clear from the graph that the role of graphene is to reduce strongly the positive dependence of gas permeability on temperature, and even cause an inversion of trend, in the case of CO_2_. These data appear for the first time and indicate that graphene nanoplatelets hinder the polymer mobility and flexibility. Indeed, it is known that the strong increase of gas diffusivity and permeability observed in polymers at increasing temperature is mostly due to the thermally-enhanced polymer chain flexibility and mobility, which makes the diffusive jumps of gas molecules more frequent. When increasing amounts of graphene are added, up to 5 wt %, to PPO, the thermal activation of diffusivity and permeability are strongly inhibited. In particular, adding 5 wt % of graphene to PPO reduces the relative increase of permeability (corresponding to a 30 °C increase) from 0.7 to 0.2 for N_2_, and from 0.47 to 0.36 for He. The relative dependence of CO_2_ permeability in pure PPO is much weaker, for the reasons discussed above, and numerically is expressed by a value of +0.13, which becomes −0.17 when the sample contains 5 wt % graphene. At graphene loadings higher than 5 wt %, the effect discussed above is partially lost, possibly because the graphene nanoplatelets start to form aggregates and lose part of their ability to immobilize the polymer.

In Figure 9b, we reported the relative selectivity variation in the temperature range inspected. The behavior is extremely clear and indicates that, for all the gases considered, the addition of even small amounts of graphene affects positively the selectivity, in all types of separation. For He/CO_2_ selectivity, favored by temperature, graphene further enhances this trend. For CO_2_/N_2_ separations, unfavored by temperature, the negative effect of a temperature increase seems mitigated by graphene addition. For He/N_2_ the selectivity starts to increase, rather than decrease, with temperature, after graphene addition. Such trends are a natural consequence of the permeability trend previously discussed, and indicate that graphene can have a marked, and beneficial, effect on the polymer mobility, which reduces the negative effects of temperature on selectivity. Indeed, at higher temperatures, the polymer becomes more flexible and loses part of its size selectivity. The addition of graphene in small amounts seems to mitigate such trend, due to the peculiar, thin and long, shape of graphene platelets which hinder polymer mobility and the loss of discriminating ability. Such effect is totally consistent with what observed when adding graphene to other glassy polymers prone to ageing: it was shown that graphene reduces ageing via the same mechanism, i.e., by acting as a physical constraint, or stabilizer, of the polymer matrix [35].

### 3.6. Comparison with Other Polymers

**Figure 10:**
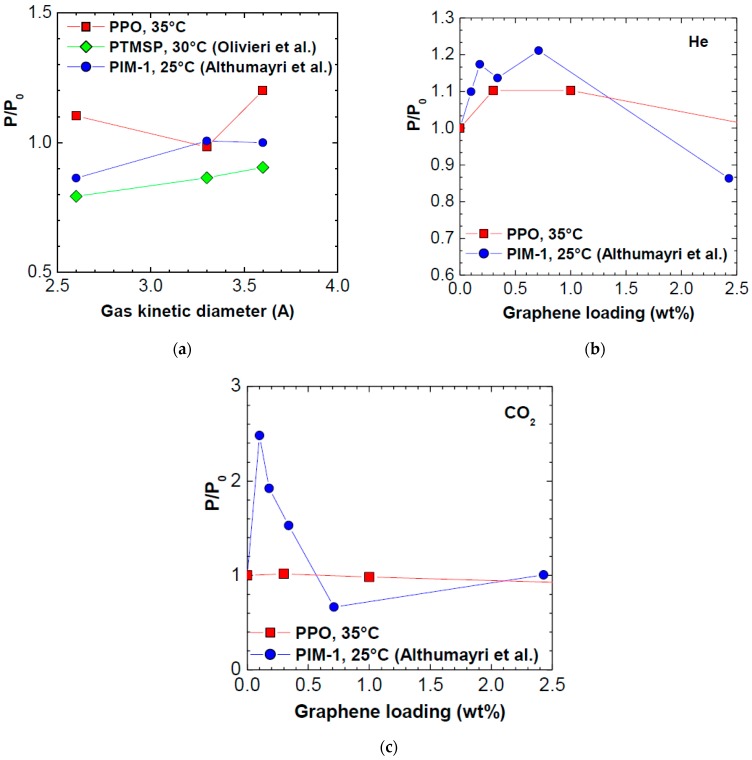
(**a**) Permeability variation induced by graphene addition, as a function of the gas kinetic diameter, in three glassy polymers: PPO (this work), PTMSP [35] and PIM-1 [33]. The weight fraction of graphene added was 1 wt % in the case of PPO and PTMSP; 0.71 wt % in the case of PIM-1. He permeability variation (**b**); and CO_2_ permeability variation (**c**) after addition of graphene in PPO (this work) and PIM-1 versus graphene loading.

The permeability variations observed with graphene have comparable order of magnitude as the ones obtained by adding 1 wt % of graphene into other glassy polymers, namely PTMSP [35] and PIM-1 [33]. In the case of PTMSP/graphene, the nominal amount of graphene in the composite membrane was 1 wt %, and the procedure used to fabricate the membrane was exactly the same used for PPO in this work, as well as the initial features of the graphene added [35]. In the case of PIM-1, the graphene content was varied between 0.1% and 2.43%, and the graphene was exfoliated in situ from graphite in the polymer solution, after a long sonication of 84 h [33]. Due to this process, it is expected that the graphene particles in such case have a smaller aspect ratio than the ones used in this work. The data of permeability variation in the various polymers inspected, after addition of 1 wt % of graphene in the case of PPO and PTMSP, and of 0.71 wt %, in the case of PIM-1, are reported in Figure 10a. Data were obtained at different temperatures in the range 25–35 °C. It can be noticed that the order of magnitude of permeability variation is the same in all polymers inspected, and the trend is increasing with increasing kinetic dimeter, indicating that the permeability of larger gases is enhanced more (or reduced less) than that of smaller gases. This could be because, for large gases, even a small adjustment of the internal free volume associated to graphene addition can make a big difference in the permeability value. It must be noted that both PIM-1 and PTMSP have much higher permeability values than PPO, and that PTMSP has a higher free volume than PPO. Plus, both such polymers, as far as the He/CO_2_ separation is concerned, are CO_2_-selective, rather than He-selective: this is due to their high free volume, and to a strong impact of the solubility on the separation. In Figure 10b,c, we report, for comparison, the trend of permeability variation for two gases, He and CO_2_, in the PPO of this work, and in PIM-1. We can notice that, in the case of helium, no significant differences between the two polymers are observed.

When it comes to CO_2_ (and similar results are noticed for N_2_), that is a slightly larger molecule, the situation changes strongly: while in PPO the CO_2_ permeability is not much affected by graphene, in the loading range inspected, marked increases of permeability are observed at very low loadings (0.1 wt %) in PIM-1. One can say that such difference can be due to the, probably, smaller aspect ratio of the graphene used in the paper by Althumayri et al., [33]. Furthermore, a significant difference may also arise from the fact that PPO is a size selective polymer, with He/CO_2_ selectivity higher than 1, while PIM-1 has a He/CO_2_ selectivity much smaller than unity.

The comparison with other polymers case indicates that, as far as graphene addition to gas separation polymers is concerned, it seems that is advisable to work in the low loading range to retain the beneficial effects. The quantitative enhancement obtained are still limited, but there is large room for improvement by working on the morphological aspects, i.e., the aspect ratio of graphene, which could be optimized by varying the preparation method. Furthermore, the effect of a surface chemistry modification of graphene platelets on the final composite properties has not been studied yet: chemistry affects the solubility contribution to permeability, but also the adhesion and morphology of the composite material. Both aspects will be investigated in future works.

## 4. Conclusions

We fabricated mixed matrix membranes based on PPO and increasing amounts of few layer graphene, from 0.3 to 15 wt %, and tested them for the permeability of He, N_2_ and CO_2_ at 35 and 65 °C.

In general, the best effect of graphene addition is observed when the loadings are small, below 1%, and this was attributed to the fact that, at high filler loading, the effect of increasing the tortuosity prevails on the effect of enhancing the polymer chain distribution. Such aspect is in agreement with previous findings on other glassy polymer filled with graphene. The selectivity for He/CO_2_ and He/N_2_ is also positively affected by addition of graphene, and the effect is observed at both 35 and 65 °C. Furthermore, the graphene addition allows adjusting the thermal dependence of permeability and selectivity, generally improving it, by acting as a physical constraint to the relaxation of polymer chains which compromises size selectivity at high temperatures.

The permeability trend follows closely the diffusivity one, confirming the validity of the solution-diffusion mechanism, and that solubility of gases is less strongly affected than diffusivity by graphene addition, for all gases except helium, which might be adsorbed onto graphene insertions.

These preliminary results suggest that small additions of graphene to PPO can enhance the permselectivity, as it does in other glassy polymers. The effect could be quantitatively improved by optimizing the aspect ratio of particles, and testing chemical modification of graphene.

## Figures and Tables

**Table 1:** List of the composite membranes produced and tested.

Graphene Type	Graphene wt % in Polymer (g/100 g of PPO)	Treatment	Sonication Time	Stirring Time	Thickness (m)
XT7	0.3	Heating at 200 °C under vacuum for 1 d	60 min	1 d	41 ± 2.1
XT6 ^a^	1		15 min		82 ± 3.9 72 ± 4.2
XT6	5				88 ± 2.6
XT6	15				55 ± 4.6

^a^ Two different membranes at 1% of XT6 were produced. The first, 82 μm thickness, was tested at 35 °C, the second, 72 μm thickness, was tested at 65 °C.

**Table 2:** Permeability of the various gases in PPO and composite membranes.

Permeability at 35 °C, Barrer	PPO	PPO/0.3 wt % Graphene XT7	PPO/1 wt % Graphene XT6	PPO/5 wt % Graphene XT6	PPO/15 wt % Graphene XT6
He	78 ± 3.8	86 ± 4.2	86 ± 4.1	68 ± 2.0	38 ± 3.2
N_2_	3.0 ± 0.2	3.5 ± 0.2	3.6 ± 0.2	2.8 ± 0.1	1.8 ± 0.2
CO_2_	61 ± 2.0	62 ± 2.9	60 ± 2.9	51 ± 1.5	27 ± 2.3
**Permeability at 65 °C, Barrer**					
He	114 ± 5.0	-	116 ± 6.7	81.0 ± 2.4	51.6 ± 4.4
N_2_	5.00 ± 0.4	-	4.64 ± 0.3	3.31 ± 0.1	-
CO_2_	69.3 ± 2	-	61.9 ± 3.6	42.3 ± 1.2	27.6 ± 2.4

**Table 3:** Ideal selectivity in PPO and composite membranes.

Ideal Selectivity at 35 °C	PPO	PPO/0.3 wt % Graphene XT7	PPO/1 wt % Graphene XT6	PPO/5 wt % Graphene XT6	PPO/15 wt % Graphene XT6
He/CO_2_	1.28	1.39	1.43	1.33	1.41
He/N_2_	26.0	24.6	23.9	24.3	21.1
CO_2_/N_2_	20.3	17.7	16.7	18.2	15.0
**Ideal Selectivity at 65 °C**	**PPO**				
He/CO_2_	1.65	-	1.88	1.92	1.87
He/N_2_	22.9	-	25.0	24.5	-
CO_2_/N_2_	13.9	-	13.3	12.8	-

**Table 4:** Diffusivity of the various gases in PPO and composite membranes.

Diffusivity at 35 °C, cm^2^/s	PPO	PPO/0.3 wt % Graphene XT7	PPO/1 wt % Graphene XT6	PPO/5 wt % Graphene XT6	PPO/15 wt % Graphene XT6
He	(5.0 ± 0.5) × 10^−6^	(7.8 ± 0.8) × 10^−7^	(3.6 ± 0.3) × 10^−6^	(10 ± 0.6) × 10^−7^	(4.4 ± 0.8) × 10^−7^
N_2_	(4.3 ± 0.4) × 10^−8^	(5.5 ± 0.5) × 10^−8^	(4.6 ± 0.4) × 10^−8^	(3.7 ± 0.2) × 10^−8^	(1.2 ± 0.2) × 10^−8^
CO_2_	(6.3 ± 0.6) × 10^−8^	(5.5 ± 0.5) × 10^−8^	(6.2 ± 0.6) × 10^−8^	(5.1 ± 0.3) × 10^−8^	(3.0 ± 0.5) × 10^−8^
**Diffusivity at 65 °C, cm^2^/s**					
He	(1.5 ± 0.2) × 10^−5^	-	(2.2 ± 0.3) × 10^−6^	(6.9 ± 0.4) × 10^−6^	(1.5 ± 0.7) × 10^−7^
N_2_	(1.1 ± 0.1) × 10^−7^	-	(7.1 ± 0.8) × 10^−8^	(7.2 ± 0.4) × 10^−8^	-
CO_2_	(1.4 ± 0.1) × 10^−7^	-	(1.4 ± 0.2) × 10^−7^	(9.5 ± 0.6) × 10^−8^	(6.7 ± 1.1) × 10^−8^

## Supplementary Materials

The supplementary materials are available online at http://www.mdpi.com/2073-4360/10/2/129/s1.
