# Supplementary material for: Permeability and Selectivity of PPO/Graphene Composites as Mixed Matrix Membranes for CO2 Capture and Gas Separation
Source: Polymers (Basel). 2018 Jan 29;10(2):129. doi: 10.3390/polym10020129 (PMC6414883; doi:10.3390/polym10020129)
Supplement: Supplementary file 1 [file polymers-10-00129-s001.docx]

**Supplementary Information**

Permeability and selectivity of PPO/graphene composites as mixed matrix membranes for CO_2_ capture and gas separation.

Riccardo Rea ^1^, Simone Ligi ^2^, Meganne Christian ^3^, Vittorio Morandi ^3^,
Marco Giacinti Baschetti ^1^, Maria Grazia De Angelis ^1,^*

^1^ Dipartimento di Ingegneria Civile, Chimica, Ambientale e dei Materiali (DICAM), Università di Bologna, Via Terracini 28, 40131 Bologna, Italy; riccardo.rea3@unibo.it (R.R.); marco.giacinti@unibo.it (M.G.B.)

^2^ Graphene XT s.r.l., 40131 Bologna, Italy; [simone.ligi@graphene-xt.com](mailto:simone.ligi@graphene-xt.com)

^3^ CNR-IMM Section of Bologna, via Gobetti, 101-40129 Bologna, Italy; christian@bo.imm.cnr.it (M.C.); morandi@bo.imm.cnr.it (V.M.)

***** Correspondence: [grazia.deangelis@unibo.it](mailto:grazia.deangelis@unibo.it) (M.G.D.A); Tel.: +39-051-2090410

|  |
| --- |

| 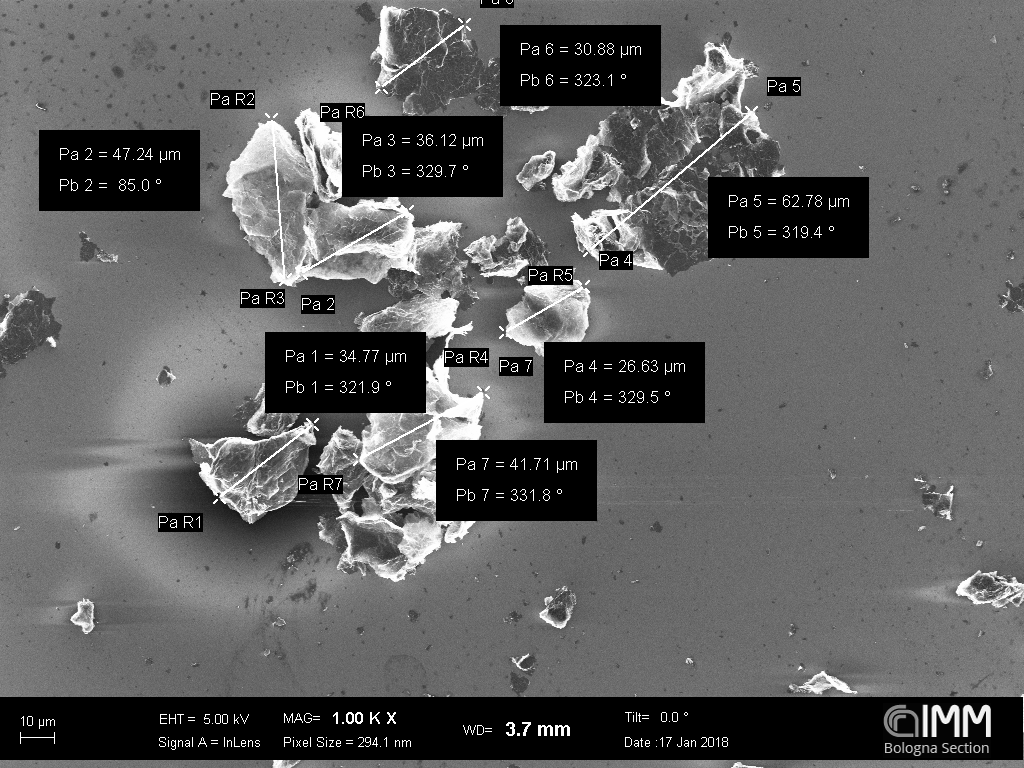 |
| --- |
|  |
| **Figure S1**: SEM images of a sample of Graphene XT 7 dispersed in water and sonicated for 10 min. |

|  |
| --- |

| **** |
| --- |
| **** |
| **Figure S2**: SEM images of a sample of Graphene XT 7 dispersed in water and sonicated for 15 h. |
